# Complex Intrahepatic Lithiasis: A Case Report of Combined Treatment With Surgical Exploration of the Bilioenteric Anastomosis and Laser Lithotripsy by Cholangioscopy

**DOI:** 10.7759/cureus.45225

**Published:** 2023-09-14

**Authors:** Marcos Eduardo Lera dos Santos, João Guilherme Ribeiro Jordão Sasso, Estela R Figueira, Victor L De Oliveira, Arthur Youssif Mota Arabi, José Donizeti Meira Júnior, Nathalia Camin Calixto Sarroche da Silva, Diogo Turiani De Moura, José Jukemura, Eduardo Guimarães De Moura

**Affiliations:** 1 Department of Gastroenterology, Hospital das Clínicas da Faculdade de Medicina da Universidade São Paulo, São Paulo, BRA; 2 Department of Gastroenterology, Gastrointestinal Endoscopy Service, Hospital das Clínicas da Faculdade de Medicina da Universidade de São Paulo, São Paulo, BRA; 3 Department of Gastroenterology, Facultad de Medicina de la Universidad de São Paulo, São Paulo, BRA; 4 Department of Gastroenterology, Gastrointestinal Endoscopy Service, Hospital das Clínicas da Faculdade de Medicina da Universidade São Paulo, São Paulo, BRA; 5 Department of Gastroenterology, Hospital das Clínicas da Faculdade de Medicina da Universidade de São Paulo, São Paulo, BRA

**Keywords:** cholangitis, endoscopic retrograde cholangiopancreatography (ercp), biliary-enteric anastomosis, intrahepatic lithiasis, hepatolithiasis, cholangioscopy

## Abstract

Intrahepatic lithiasis, or hepatolithiasis, is an endemic disease in southeast Asia, although, with immigration from Eastern countries, the incidence of this pathology is rising worldwide. The Latin American experience demonstrates morbidity and mortality compatible with other Western countries, but minimally invasive procedures are lacking. We demonstrate a case of a combined surgical and endoscopic approach for stone clearance.

We present a case of a 47-year-old female patient with biliary enteric anastomosis to treat recurrent pyogenic cholangitis resulting from intrahepatic lithiasis. The patient was admitted to the emergency room, presented with a new episode of cholangitis, and submitted to transcutaneous hepatobiliary drainage. The multidisciplinary approach, including the endoscopic and surgical teams, successfully performed the stone clearance with laser lithotripsy and stone removal by open access. The postoperative period was uneventful, and the patient did not present any sign of recurrence after one year.

A combined surgical and endoscopic approach achieved short-term clinical and technical success in this novel case. Moreover, individualizing cases requiring open surgical access is feasible, which allows a combined endoscopic approach with safety.

## Introduction

Intrahepatic lithiasis (IHL) or hepatolithiasis is a disease of great repercussion in eastern countries, endemic in Southeast Asia, with a relative incidence reaching 45% in some regions of China and 20% in Taiwan when comparing all causes of gallstones. Nevertheless, the IHL incidence and importance increased significantly in Western countries after intense immigration from Asia [1, 2].

Despite being a benign disease, IHL can present multiple complications, such as recurrent pyogenic cholangitis, liver abscesses, secondary biliary cirrhosis, and cholangiocarcinoma. This disease of high morbidity and mortality was observed in the nineties in Japan in a follow-up of four to 10 years of the patients. A recurrence rate of 29.6% and a mortality rate of 10.6% were evidenced [2,3]. In the Latin American experience, the 30-day mortality is around 0.7%, and the morbidity is 30% after surgery [4].

Thus, minimally invasive therapeutics for treating IHL are on the rise, such as biliary tract exploration via the left hepatic duct, percutaneous transhepatic drainage, per-oral cholangioscopy, and cholangioscopy associated with laparoscopic and robotic procedures [5-8]. We describe a case of laser lithotripsy via biliary-enteric anastomosis utilizing cholangioscopy in a combined surgical and endoscopic procedure.

## Case presentation

We hereby present the case of a 47-year-old woman with IHL (Video 1).

**Video 1:** Case presentation This video describes the case and demonstrates the radiological aspects preceding the surgery.

She had a history of multiple choledocholithiasis and recurrent pyogenic cholangitis, being subjected to several endoscopic retrograde cholangiopancreatographies (ECRP) for biliary tract clearance and placement of biliary stents. Despite all treatments, she developed biliary stricture at the hepatic hilum and underwent biliary-enteric anastomosis (BEA) surgery with Roux-en-Y anastomosis in an external gastrointestinal unit. Five years later, she developed BEA stricture, and new intrahepatic calculi were formed, leading to recurrent pyogenic cholangitis and liver abscesses. Furthermore, the patient was subjected to five ERCPs and, finally, one percutaneous transhepatic drainage of the biliary tract in one episode of cholangitis Tokyo II [9]. The case was discussed in a multidisciplinary conference, and the patient was submitted to a surgical and endoscopic combined approach. The initial blood parameters are demonstrated in Table 1.

**Table 1:** Initial blood parameters. AST: alanine aminotransferase; ALT: aspartate aminotransferase

Parameter	Value	Reference range
Hemoglobin	12 g/dL	12.0 - 16.0 g/dL
Leukocytes	9800 cell/mm^3^	4500 - 11000 cell/mm^3^
Platelets	217,000 /mm^3^	150 - 400 x10^9^/L
Total bilirubin	5.65 mg/dL	0.3 - 1.0 mg/dL
Direct bilirubin	5.31 mg/dL	<0.3 mg/dL
AST	80 U/L	8 - 48 U/L
ALT	174 U/L	7 - 55 U/L

The intraoperative cholangioscopy performed to remove intrahepatic calculi combined with a BEA evaluation is showcased in Video 2.

**Video 2:** Combined surgical and endoscopic procedure This video demonstrates endoscopic and surgical procedures, focusing on the cholangioscope view of the biliary tree and gadgets for stone removal.

**Figure 1:**
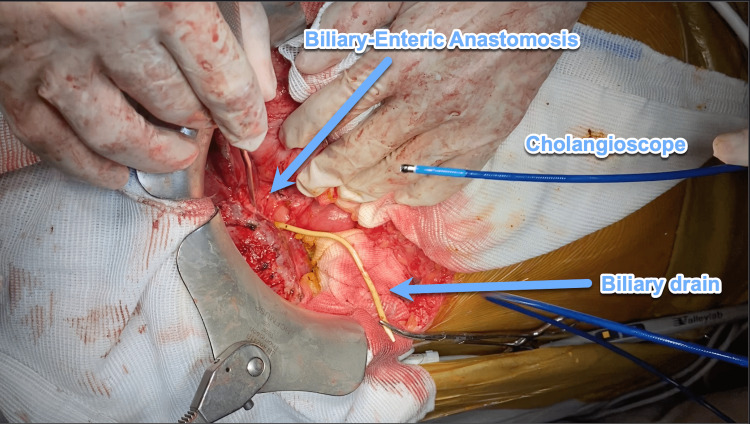
The surgical, open-access view of the biliary anastomosis, percutaneous biliary drain, and cholangioscope.

First, the patient was subjected to a surgical procedure in which the previous BEA stricture was found and opened (Figure 1).

**Figure 2:**
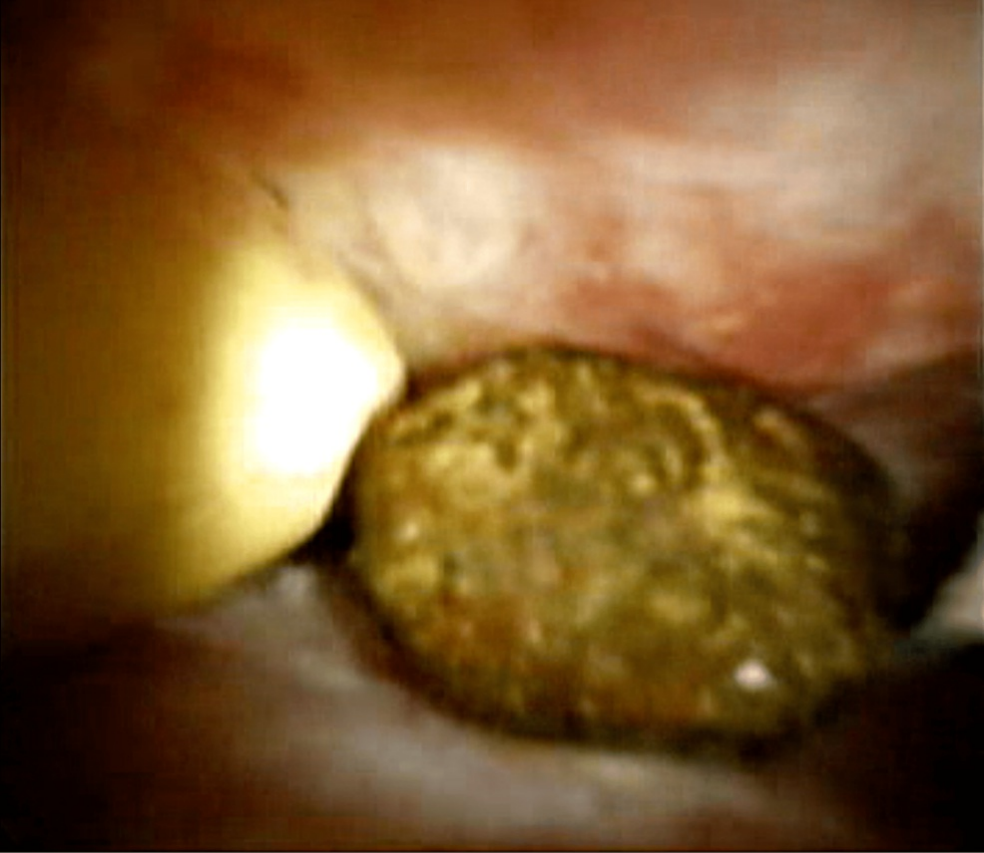
Cholangioscope view of hepatic lithiasis This image demonstrates a cholangioscope view of calculi in the biliary tree.

**Figure 3:**
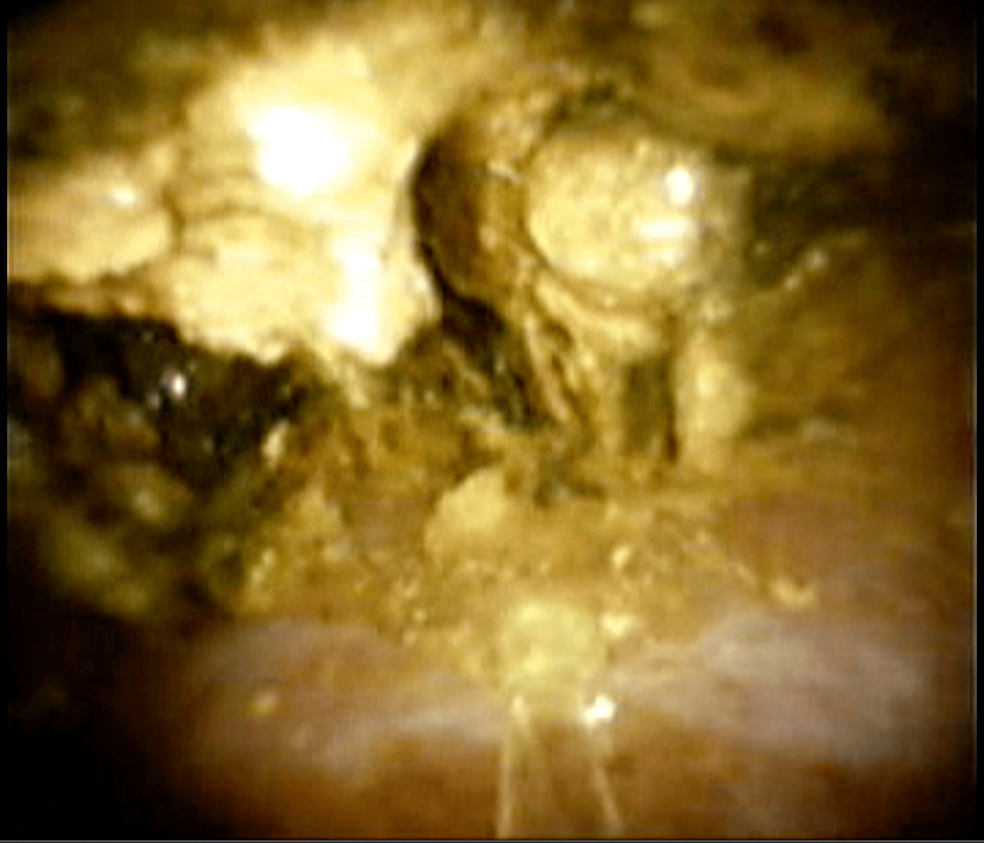
Laser lithotomy by cholangioscope.

Then, a sterile cholangioscope was inserted through the biliary tract, and it was possible to reach the thin biliary branches due to the small diameter of the instrument. Numerous biliary stones were found (Figure 2) and removed employing adjuvant devices such as laser lithotripsy (Figure 3) and endoscopic baskets, as shown in the video (Video 2).

The use of intraoperative cholangiography showed excellent immediate results. The postoperative period was uneventful, and the patient was discharged in good health. After one year, the patient has not presented with jaundice or any other sign of recurrence.

## Discussion

Intrahepatic lithiasis, or hepatolithiasis, is a rare disease in Western countries. It occurs when stones are identified within the bile ducts, proximal to the confluence of the right and left biliary ducts [10]. Despite being a benign disease, IHL can present multiple complications, such as repetitive cholangitis, liver abscesses, liver atrophy, hepatic abscesses, portal hypertension, and secondary biliary cirrhosis [8, 10]. Although rare, some cases of intrahepatic cholangiocarcinoma are also related to long-term hepatolithiasis [10]. As a pathology of great repercussion previously in Asia and now worldwide, IHL is a disease with various presentations and often complex treatments. The clinical presentation is essential to defining the best approach. For example, in children, most of the cases are asymptomatic in the right lobe and thus should be observed until clinical symptoms begin, as demonstrated in a retrospective cohort study in Beijing Children's Hospital with 106 pediatric patients [11].

When treating IHL, some different approaches can be used. In treating intrahepatic lithiasis, it must be considered that the main goals should be to control infections, remove stones, and attempt to minimize the risk of developing cholangiocarcinoma [10]. The surgical approach is still the gold standard for treating IHL; hepatic resection is the primary option for IHL treatment once it can remove the stones and create reasonable biliary drainage, and it is associated with a low recurrence rate. Hepatectomy alternatives are usually performed in patients with suspected cholangiocarcinoma, unilobar lithiasis, liver atrophy, and those with symptoms like cholangitis [2,10].

Despite that, it is known that the surgical procedure is not free of complications, especially in cases of liver resection. Surgical complications play an essential role in the definition of the management approach, considering that the surgical procedure may be associated both with surgical issues (such as biliary leakage, intra-abdominal abscess, and bleeding) and clinical complications (such as pneumonia, deep vein thrombosis (DVT), and even sepsis) [2]. In this scenario, the use of less invasive techniques, such as endoscopic intervention, is increasing, including as an intraoperative tool to complement surgical treatment, once it allows performing direct lithotomy or lithotripsy with more safety and efficiency [2,12,13].

The initial open surgical access presented significant morbidity and mortality [3]. The procedure is safer with new technologies such as robotic and laparoscopic procedures. It has less recurrence, as demonstrated in a retrospective cohort with 33 centers and 273 patients submitted to laparoscopic or robotic resection for hepatic lithiasis. The two procedures demonstrated similar results for mortality and morbidity. A robotic procedure was statistically significantly associated with less blood loss and less conversion to open surgery [7].

Furthermore, when we evaluated left-sided IHL, a meta-analysis demonstrated new options rather than resection, such as cholangioscopy via the left hepatic duct orifice. The procedure involves a percutaneous trajectory performed and matured for a cholangioscope passage [14]. This procedure and a common bile duct incision were compared, demonstrating that cholangioscopy has similar success in stone clearance with shorter postoperative hospitalization [15]. Likewise, the per-oral cholangioscopy had been used in case reports, with promising results, especially in those cases of refractoriness to ERCP [16].

We demonstrated a complex case of IHL with previous BEA surgery and hepatic drainage. In our case, we chose to perform an intraoperative cholangioscopy because the surgery would be performed; nevertheless, as the BEA stricture needed reconstruction, the cholangioscopy should help with the clearance of IHL, as in other related cases. In this novel case, we achieved technical and clinical success, demonstrating that this is a feasible option and that the association of procedures can be done in select cases.

## Conclusions

In the present case, the combined surgical and endoscopic approach was adopted after the evaluation of a multidisciplinary team. The procedure was performed without complications, with technical and short-term clinical success. Thus, there are advantages to a combined approach in terms of stone clearance and safety when open surgical exploration is needed. Treating IHL remains a challenge, and there is a need for minimally invasive procedures with long-term clinical success.
